# Effect of methylprednisolone treatment on COVID-19: An inverse probability of treatment weighting analysis

**DOI:** 10.1371/journal.pone.0266901

**Published:** 2022-06-17

**Authors:** Lorenzo Porta, Sih-Shiang Huang, Chen Wei, Chin-Hua Su, Wan-Ting Hsu, Wang-Huei Sheng, Chien-Chang Lee

**Affiliations:** 1 School of Medicine and Surgery, Department of Emergency Medicine, Università degli studi di Milano Bicocca, Milano, Italy; 2 Department of Emergency Medicine, National Taiwan University Hospital, Taipei, Taiwan; 3 Department of Medicine, Harvard Medical School, Boston, MA, United States of America; 4 Department of Internal Medicine, Stanford Health Care, Stanford, CA, United States of America; 5 Department of Epidemiology, Harvard T.H. Chan School of Public Health, Boston, MA, United States of America; 6 Department of Internal Medicine, National Taiwan University Hospital, Taipei, Taiwan; 7 The Centre for Intelligent Healthcare, National Taiwan University Hospital, Taipei, Taiwan; 8 Byers Center for Biodesign, Stanford University, Stanford, CA, United States of America; The University of Mississippi Medical Center, UNITED STATES

## Abstract

**Objectives:**

While corticosteroids have been hypothesized to exert protective benefits in patients infected with SARS-CoV-2, data remain mixed. This study sought to investigate the outcomes of methylprednisone administration in an Italian cohort of hospitalized patients with confirmed SARS-CoV-2 infection.

**Methods:**

Patients with confirmatory testing for SARS-CoV-2 were retrospectively enrolled from a tertiary university hospital in Milan, Italy from March 1st to April 30th, 2020 and divided into two groups by administration of corticosteroids. Methylprednisolone was administered to patients not responding to pharmacological therapy and ventilatory support at 0.5-1mg/kg/day for 4 to 7 days. Inverse probability of treatment weighting (IPTW) was used to adjust for baseline differences between the steroid and non-steroid cohorts via inverse probability of treatment weight. Primary outcomes included acute respiratory failure (ARF), shock, and 30-day mortality among surviving patients.

**Results:**

Among 311 patients enrolled, 71 patients received steroids and 240 did not receive steroids. The mean age was 63.1 years, 35.4% were female, and hypertension, diabetes, heart disease, and chronic pulmonary disease were present in 3.5%, 1.3%, 14.8% and 12.2% respectively. Crude analysis revealed no statistically significant reduction in the incidence of 30-day mortality (36,6% vs 21,7%; OR, 2.09; 95% CI, 1.18–3.70; p = 0.011), shock (2.8% vs 4.6%; OR, 0.60; 95% CI = 0.13–2.79; p = 0.514) or ARF (12.7% vs 15%; OR, 0.82; 95% CI = 0.38–1.80; p = 0.625) between the steroid and non-steroid groups. After IPTW analysis, the steroid-group had lower incidence of shock (0.9% vs 4.1%; OR, 0.21; 95% CI,0.06–0.77; p = 0.010), ARF (6.6% vs 16.0%; OR, 0.37; 95% CI, 0.22–0.64; p<0.001) and 30-day mortality (20.3% vs 22.8%; OR 0.86; 95% CI, 0.59–1.26 p = 0.436); even though, for the latter no statistical significance was reached. Steroid use was also associated with increased length of hospital stay both in crude and IPTW analyses. Subgroup analysis revealed that patients with cardiovascular comorbidities or chronic lung diseases were more likely to be steroid responsive. No significant survival benefit was seen after steroid treatment.

**Conclusions:**

Physicians should avoid routine methylprednisolone use in SARS-CoV-2 patients, since it does not reduce 30-day mortality. However, they must consider its use for severe patients with cardiovascular or respiratory comorbidities in order to reduce the incidence of either shock or acute respiratory failure.

## Introduction

Since its outbreak in December 2019 in Wuhan, China, the novel coronavirus (SARS-CoV-2) has rapidly become the worst pandemic of the last century [1–3]. To date, SARS-CoV-2 has infected more than 300 million people, causing more than 5 million deaths [4]. Sharing similar genetic constitution and lung pathophysiology with the first SARS-CoV [5], this novel disease has multiple clinical manifestations, ranging from mild symptoms to acute respiratory distress syndrome (ARDS), shock and multiorgan dysfunction [6–9]. At the current time, no treatment has been proven to be effective [6]. Even though remdesivir, hydroxychloroquine and azithromycin have been proposed as possible candidates, strong clinical evidence lacked to support their widespread use [10–13].

Corticosteroids may have a role in the treatment of Covid-19. In particular, due to their anti-inflammatory and anti-fibrotic effects, they may reduce the severity of cytokine release syndrome and prevent progression to pulmonary fibrosis, a severe complication of SARS-CoV-2 disease [14–17]. This hypothesis has been supported by some previous studies [18], and a multicenter randomized controlled trial has reported that early administration may reduce all-cause mortality and duration of mechanical ventilation for ARDS patients [19]. Corticosteroids may not only reduce mortality in sepsis patients [20], but also have beneficial effects in the treatment of Covid-19, especially when administered in the early stages of the disease [21, 22]. Nevertheless, the data is mixed, with some reports supporting their use, especially in severe cases of ARDS [23, 24], and other studies reaching opposite conclusions, arguing that routine administration of corticosteroid can increase mortality [25–28]. To provide further clarity on the potential role of corticosteroids in Covid-19, we used propensity score analysis to study the effect of methylprednisolone on outcomes in a cohort of Italian patients.

## Methods

### Study design and participants

A retrospective cohort study of SARS-CoV-2 patients was conducted using a cohort of patients hospitalized in a tertiary university hospital in Milan, Italy. SARS-CoV-2 data were retrospectively collected and de-identified from databases of electronic medical records. The study was approved by the Research Committee of Università degli studi di Milano-Bicocca, Milan, Italy. Medical records of patients admitted to the Emergency ward of Ospedale Maggiore Niguarda, Milan, Italy, from the 1st of March to the 30th of April 2020 were anonymously collected. Due to the retrospective observational nature of our study, the impracticability of obtaining informed consent in debilitated semi-intensive patients and the use of de-identified data, waiver for informed consent was granted. Their data were screened for the following inclusion criteria: 1) Age greater than 18 years old; 2) Confirmation of SARS-CoV-2 pneumonia with a positive swab at admission and typical CT chest pattern of ground-glass opacities and bilateral patchy shadowing. All SARS-CoV-2 patients admitted with a concomitant diagnosis (ie, bowel obstruction, syncope, stroke) were excluded. Glucocorticoids were not prescribed routinely, due to the lack of published data on the issue, but were reserved for severe patients (defined as those with a deteriorating oxygen saturation, not responding to ventilation support), based on the opinion of the physician. Low dose methylprednisolone was used for 4 to 7 days at a dose of 0.5–1 mg/kg/day.

### Definitions and outcomes

The following covariates were extracted from the dataset: age, sex, ethnicity, race, smoking status, vital signs including temperature, peripheral oxygen saturation (SpO2), heart rate, respiratory rate (RR), blood pressure (BP), and laboratory results including white blood cell count (WBC), hemoglobin, estimated glomerular filtration rate (eGFR), alanine aminotransferase (ALT), aspartate aminotransferase (AST), C-reactive protein (CRP), and procalcitonin. We also collected data on the following comorbidities: obesity, immunosuppression, active tumor, pulmonary disease, smoking status, heart disease, vasculopathy, chronic renal failure, arthritis. The outcomes studied included: acute respiratory failure, shock, all-cause mortality and length of hospital stay among surviving patients. We defined acute respiratory failure as the worsening of the general respiratory condition of the patient, necessitating endotracheal intubation and mechanical ventilation.

### Statistical analysis

The baseline characteristics of the enrollees were described and compared among steroid-treated and steroid-untreated groups. Categorical variables were presented as a frequency and percentage and were compared between cases and controls using the χ2 test. Continuous variables were presented as median and IQR and were compared using the Mann–Whitney U test. To adjust for baseline differences in patient comorbidity and disease severity, inverse probability of treatment weighting (IPTW) was employed. This method was chosen over propensity score (PS) matching because the number of patients between the IHCA and non-IHCA cohorts was highly imbalanced. In IPTW—essentially the inverse of the PS—patients are weighted in inverse proportion to their probability of receiving treatment; thus, those more likely to receive treatment are weighted lower than those less likely. This method creates weighted individual samples, which avoids the loss of unmatched treated patients that might otherwise occur in PS matching. By creating weighted samples of treatment and control individuals with balanced baseline covariates, IPTW mimics a randomized control trial. We created a PS including 23 potential predictors that reflects the conditional probability of receiving steroid treatment versus controls. To visualize the balance of covariates after IPTW, we plotted the standardized mean differences of all covariates before and after IPTW (S1 Fig). In online S1 Table, we report the c-statistics of the PS model, component variables and the respective weights of the component variables. Briefly, the covariates are: age, gender, comorbidities, presenting vital signs, laboratory results and concomitant treatment. To further assess the potential interaction effects, we performed subgroup analyses. For each study outcome, an odd ratio with associated 95% confidence interval was estimated for the IPTW samples using a weighted binary logistic regression model with a robust variance estimator. In addition, Kaplan–Meier survival plots were generated to track mortality over time for the original and IPTW cohorts. Furthermore, a series of subgroup analyses were performed to test the potential interaction between steroid treatment and the predefined clinically meaningful subgroups, including age, gender, presence of cardiovascular diseases, diabetes or obesity. A *p-*value of 0.05 or less was considered significant. All analyses were carried out using SAS V.9.3 for Windows (SAS Institute Inc, Cary, North Carolina, USA), and the data are reported in accordance with STROBE guidelines.

## Results

A total of 311 patients were eligible for the study. Of these patients, 71 received steroid treatment, while 240 received non-steroid treatment. In the study, the mean age was 63.1 years and there were 35.4% of female patients. A history of hypertension was present in 3.5% of the patients, diabetes mellitus in 1.3%, heart disease in 14.8% and chronic pulmonary disease in 12.2%.

The baseline characteristics for steroid group and non-steroid group are shown in Table 1. Steroid group patients were older (p<0.001); suffered more from diabetes (p = 0.002), immunosuppression (p = 0.004), active tumor (p = 0.017); had higher respiratory rate in the emergency department (p = 0.029) and lower diastolic blood pressure (p = 0.041). There were no significant differences in sex (p = 0.307), and the following comorbidities: hypertension (p = 0.470), chronic pulmonary disease (p = 1.000), coronary artery disease (1.000), stroke (p = 0.898) and chronic kidney disease (1.000). For the pre-treatment laboratory results of each group, white blood cell count (steroid, 18.359 10^3^/mm^3^; non-steroid, 6.386 10^3^/mm^3^; p = 0.038), hemoglobin (steroid, 13.25 g/dL; non-steroid, 13.09 g/dL; p = 0.011), hs-CPR (steroid, 8.61 mg/dL; non-steroid, 6.11 mg/dL; p = 0.008), ALT (steroid, 27.06 U/L; non-steroid, 24.07 U/L; p<0.001), Creatinine (steroid, 1.65 mg/dL; non-steroid, 1.13 mg/dL; p<0.001), and Urea (steroid, 13.16 mg/dL; non-steroid, 11.97 mg/dL; p<0.001) were higher in the steroid treatment group. No significant difference was noted for symptoms; more patients in the steroid group were concomitantly treated with Lopinavir/ritonavir (steroid, 91.5%; non-steroid, 74.6%; p = 0.004).

**Table 1:** Comparison of characteristics between patients receiving steroid treatment and controls.

	Steroid use (n = 71)	Non steroid use (n = 240)	p-value
Demographics			
Age (mean (SD))	69.92 (13.72)	61.12 (17.07)	<0.001
Sex male	50 (70.4%)	151 (62.9%)	0.307
Symptoms			
Fever	61 (85.9%)	224 (93.3%)	0.082
Cough	40 (56.3%)	144 (60.0%)	0.679
Dyspnea	25 (35.2%)	78 (32.5%)	0.777
Headache	4 (5.6%)	29 (12.1%)	0.183
Diarrhea	6 (8.5%)	33 (13.8%)	0.327
Syncope	5 (7.0%)	9 (3.8%)	0.396
Symptom duration	5.31 (3.16)	5.93 (3.66)	0.198
Comorbidity			
Hypertension	4 (5.6%)	7 (2.9%)	0.470
Diabetes	4 (5.6%)	0 (0.0%)	0.002
Active tumor	11 (15.5%)	14 (5.8%)	0.017
Immunosuppression	10 (14.1%)	9 (3.8%)	0.004
Chronic pulmonary disease	10 (14.1%)	36 (15.0%)	1.000
Coronary artery disease	9 (12.7%)	29 (12.1%)	1.000
Stroke	6 (8.5%)	17 (7.1%)	0.898
Chronic Kidney disease	5 (7.0%)	12 (5.0%)	0.713
Vital sign on presentation (mean (SD))			
Temperature	37.48 (0.30)	37.50 (0.27)	0.603
Heart rate	97.39 (5.74)	98.47 (4.14)	0.082
Respiratory rate	21.40 (2.31)	20.96 (1.13)	0.029
Systolic blood pressure	130.88 (11.28)	130.47 (6.76)	0.703
Diastolic blood pressure	75.58 (4.67)	76.46 (2.59)	0.041
Laboratory results (mean (SD))			
White blood cell (10^3^/mm^3^)	18.36 (8.93)	6.39 (3.08)	0.038
Hemoglobin concentration (g/dL)	13.25 (0.68)	13.09 (0.36)	0.011
Platelet count (10^5^/mm^3^)	1.84 (7.58)	2.00 (7.90)	0.145
Absolute neutrophil count (10^3^/mm^3^)	4.10 (1.00)	3.94 (0.72)	0.135
Absolute lymphocyte count (10^3^/mm^3^)	1,33 (2.59)	1.12 (0.43)	0.230
hs-CRP (mg/dL)	8.61 (7.28)	6.11 (6.91)	0.008
PCT (ng/mL)	1.20 (5.38)	1.01 (10.51)	0.887
Total bilirubin (mg/dL)	0.58 (0.28)	0.57 (0.36)	0.849
ALT (U/L)	27.06 (5.01)	24.07 (3.08)	<0.001
Creatinine (mg/dL)	1.65 (1.74)	1.13 (0.76)	<0.001
Blood sugar (mg/dL)	121.81 (23.62)	117.68 (13.07)	0.058
Urea nitrogen (mg/dL)	13.16 (2.87)	11.97 (1.37)	<0.001
Concomitant treatment			
Lopinavir/ritonavir	65 (91.5%)	179 (74.6%)	0.004

*Note: hs-CRP, high sensitivity C-reactive protein; PCT, procalcitonin; ALT, alanine aminotransferase.

In the crude analysis, steroid treatment was not significantly associated with reduced incidence of either shock or acute respiratory failure (ARF), however it was associated with an increase in 30-day mortality (steroid, 36.6%; non-steroid, 21.7%; OR, 2.09; 95% CI, 1.18–3.70; p = 0.011) and length of hospital stay among the survived patients (Table 2). After the application of inverse probability of treatment weighting (IPTW), we noted that the steroid-group had lower incidence of shock (steroid, 0.9%; non-steroid, 4.1%; OR, 0.21; 95% CI,0.06–0.77; p = 0.010), ARF (steroid, 6.6%; non-steroid, 16.0%; OR, 0.37; 95% CI, 0.22–0.64; p<0.001) and 30-day mortality (steroid, 20.3%; non-steroid, 22.8%; OR 0.86; 95% CI, 0.59–1.26 p = 0.436); even though, for the latter no statistical significance was reached (Table 2). Furthermore, steroid use was associated with increased duration of hospitalization both in crude and IPTW analyses.

**Table 2:** Comparison of in-hospital mortality between patients with/without steroid treatment. Survival differences were calculated as odds ratio (OR). We used the IPTW method to adjust for the potential confounding.

	Steroid treatment (n = 71)	Non-steroid treatment (n = 240)	OR (95% CI)	p-value
Crude analysis				
Shock	2 (2.8%)	11 (4.6%)	0.60 (0.13–2.79)	0.5142
Acute respiratory failure	9 (12.7%)	36 (15.0%)	0.82 (0.38–1.80)	0.6254
30-day mortality	26 (36.6%)	52 (21.7%)	2.09 (1.18–3.70)	0.0108
Length of hospital stay among the survived patients (median, 25th and 75th quartile)	10 (8–11)	7 (6–10)	NA	0.0016
IPTW-weighted analysis (n = 313) (n = 309)				
Shock	3 (0.9%)	13 (4.1%)	0.21 (0.06–0.77)	0.0096
Acute respiratory failure	21 (6.6%)	49 (16.0%)	0.37 (0.22–0.64)	0.0002
30-day mortality	63 (20.3%)	71 (22.8%)	0.86 (0.59–1.26)	0.4364
Length of hospital stay among the survived patients (median, 25th and 75th quartile)	10 (8–11)	8 (6–10)	NA	0.0014

**Fig 1:**
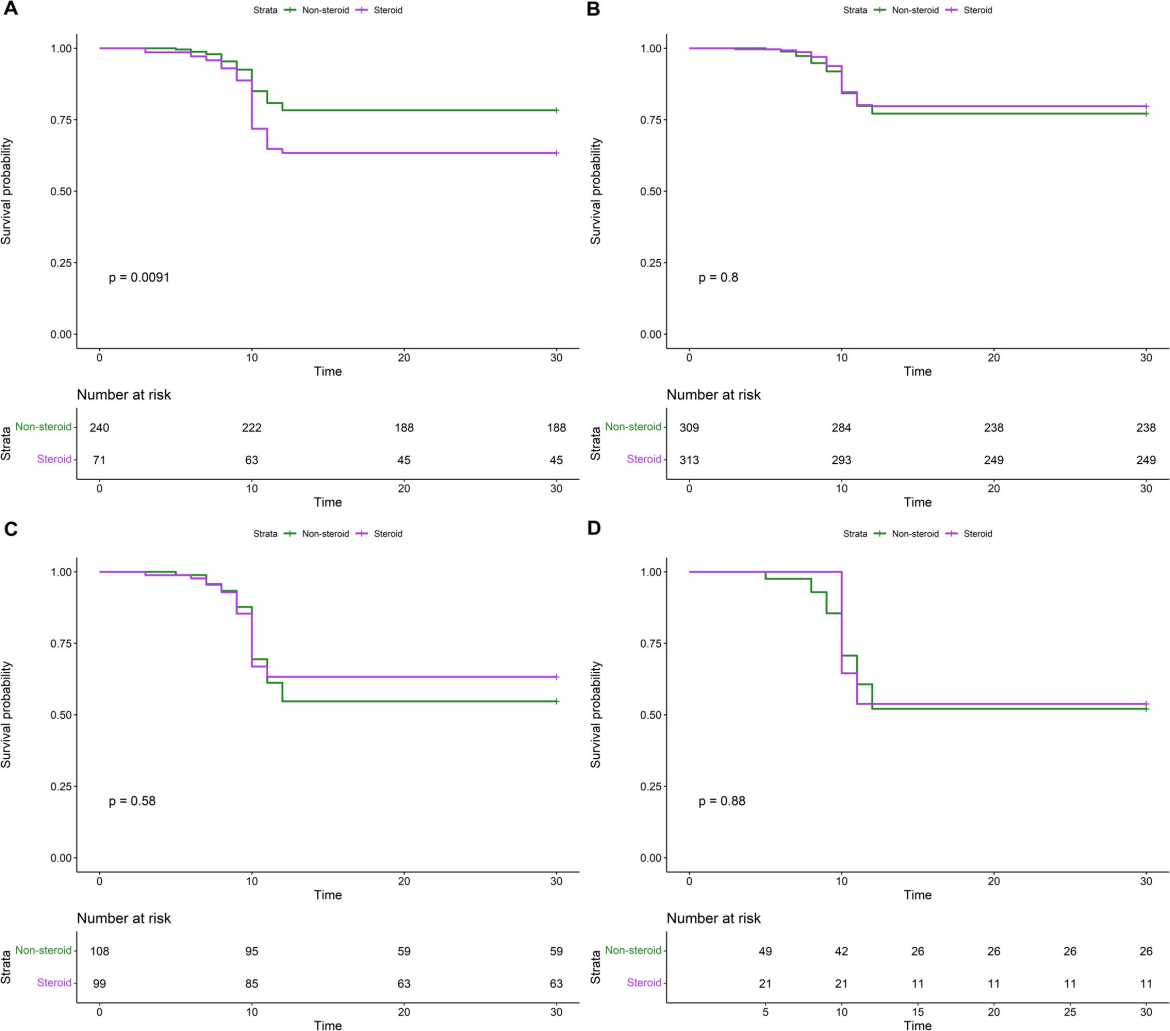
The 30-day Kaplan-Meier cumulative survival analyses between patients with steroid pretreatment versus patients without steroid pretreatment. (A) Before IPTW (B) After IPTW (C) Acute respiratory distress (Oxygen dependent) (D) Acute respiratory failure (requirement of ventilator).

Additionally, we performed subgroup analysis on post-discharge 30-day mortality after IPTW. We observed that patients with cardiovascular comorbidities or chronic lung diseases were more likely to respond to steroids (Table 3). The 30-day Kaplan-Meier survival curves are shown in Fig 1. There were no mortality differences after IPTW between the steroid and non-steroid groups (Fig 1B). When applying subgroup analyses in acute respiratory distress and acute respiratory failure patients, this persisted (Fig 1C and 1D).

**Table 3:** Subgroup analysis for 30-day in-hospital mortality after IPTW-weighting.

	Hazard ratio (95% CI)	Interaction P
Age > 75 years	0.82 (0.59–1.29)	0.9299
Age < = 75 years	0.75 (0.45–1.27)	
Male	0.90 (0.62–1.31)	0.7716
Female	0.75 (0.35–1.63)	
Cardiovascular disease*	0.53 (0.32–0.88)	0.0036
Non-cardiovascular disease	1.26 (0.79–2.03)	
Acute respiratory failure	0.92 (0.43–1.95)	0.8685
Non-acute respiratory failure	1.02 (0.69–1.51)	
Shock	1.92 (0.24–15.71)	0.6340
Non-shock	0.88 (0.62–1.64)	
Chronic lung disease	0.26 (0.11–0.60)	0.0018
No chronic lung disease	1.15 (0.78–1.67)	
Acute respiratory distress (oxygen dependent)	0.83 (0.54–1.27)	0.2012
Non-acute respiratory distress	1.15 (0.65–2.03)	

*Cardiovascular disease includes myocardial infarction, cerebrovascular disease, peripheral vascular disease and congestive heart failure.

## Discussion

In this study, we investigated the effect of short cycles of low-dose methylprednisolone in patients with SARS-CoV-2 related pneumonia. To do this, we collected data from 311 Italian patients at a tertiary referral hospital. After the IPTW analysis, we found that corticosteroid users had a lower incidence of shock and acute respiratory failure, but no reduction in 30-day mortality was evident. Furthermore, we demonstrated that corticosteroid use increased the length of hospital stay; we can hypothesize that this relationship might be due to the possible side effects of its use, such as electrolyte abnormalities, hypertension or hyperglycemia, for example. Patients with cardiovascular comorbidities or chronic lung diseases were more likely to be steroid responsive. The results of our study support the assertion that corticosteroids can be beneficial in severe patients, particularly in those with cardiovascular or respiratory comorbidities, but are not recommended for routine use, considering the risk/benefit ratio and the associated increase in hospitalization duration.

Corticosteroids, due to their anti-inflammatory and anti-fibrotic effects, have been hypothesized to have utility in the management of SARS-CoV-2 related pneumonia and ARDS via reduction of the severity of cytokine release syndrome, preventing progression to pulmonary fibrosis. However, corticosteroid use is accompanied by side effects, including immunosuppression, hyperglycemia, hypokalemia, kidney damage and altered mental state. This dichotomy of risk and benefits regarding the use of corticosteroids in SARS-CoV-2 patients could be related to the severity of the disease. In fact, while several studies have stated that the routine administration of corticosteroid can affect survival, increasing mortality [25, 26, 29], a recent multicenter, randomized controlled trial (Randomised Evaluation of COVID-19 therapy trial–RECOVERY) has reported that the early administration of corticosteroid might reduce all-cause mortality and duration of mechanical ventilation for ARDS patients [23].

The RECOVERY trial was a large trial that randomized patients hospitalized with severe Covid-19 infection to receive one (or none) of the trial treatments (including low-dose dexamethasone). The primary outcome was all-cause mortality at 28 days. The RECOVERY trial observed that low-dose dexamethasone could reduce mortality among patients receiving invasive mechanical ventilation or among those receiving oxygen without invasive mechanical ventilation. No beneficial effect was registered among those receiving no respiratory support. However, some limitations were identified. First, the dexamethasone use was found beneficial only for severe patients and could not be applied as a routine therapy. Second, at the end of the 28-day trial period around a third of patients were still hospitalized, thus, their final outcomes were not known.

There are several reasons why our results differ from those in the RECOVERY trial. Firstly, the retrospective and non-randomised nature of our study could have affected our results, due to bias we could not exclude with the analysis. Second, our cohort was composed of a population of patients, coming from the second most hit by SARS-CoV-2 country; in comparison, the British cohort studied in RECOVERY was affected later on. Third, our patients were prescribed methylprednisolone and not dexamethasone. Even though the two drugs are part of the same category; individual effects differ and could have affected the results; in fact, the benefit in mortality might have been more noteworthy over longer follow-up. However, even though some differences between the two studies are present, our results are consistent with the published data. To this end, we demonstrated that methylprednisolone had no beneficial effect in reducing mortality, however similarly to the RECOVERY trial, we observed a potential protective effect of the use of corticosteroid on acute respiratory failure and shock, especially in patients with cardiovascular or respiratory comorbidities. Our study supported a possible beneficial effect of the use of corticosteroid could be applicable to severe patients, while the routine use should be avoided.

## Conclusion

Physicians should avoid routine methylprednisolone use in SARS-CoV-2 patients, as its use is not associated with any beneficial effects in reducing 30-day mortality. However, they must consider methylprednisolone use for severe patients in order to reduce the incidence of either shock or acute respiratory failure, especially in patients with cardiovascular or respiratory comorbidities.

## Supporting information

S1 Checklist STROBE statement—Checklist of items that should be included in reports of observational studies. (PDF)

S1 Fig Standardized difference graph before and after IPTW. *Note: hs-CRP, high sensitivity C-reactive protein; SBP, systolic blood pressure; DBP, diastolic blood pressure; wbc, white blood cell count PCT, procalcitonin; HTN, hypertension; CKD, chronic kidney disease; CAD, coronary artery disease.
(TIFF)

S1 Table Empirical predictors of steroid treatment with associated odds ratios for in the propensity score model. *Note: CKD, chronic kidney disease; hs-CRP, high sensitivity C-reactive protein; PCT, procalcitonin.
(DOCX)
