# Supplementary material for: Effect of methylprednisolone treatment on COVID-19: An inverse probability of treatment weighting analysis
Source: PLoS One. 2022 Jun 17;17(6):e0266901. doi: 10.1371/journal.pone.0266901 (PMC9205494; doi:10.1371/journal.pone.0266901)
Supplement: S1 Table — *Note: CKD, chronic kidney disease; hs-CRP, high sensitivity C-reactive protein; PCT, procalcitonin. (DOCX) [file pone.0266901.s003.docx]

**Supplementary eTable 1.** Empirical predictors of steroid treatment with associated odds ratios for in the propensity score model

| Covariates | OR (95%CI) |
| --- | --- |
| Demographic | |
| Age | 1.18 (0.84- 1.65) |
| Male | 0.97 (0.45- 2.12) |
| Comorbidity | |
| Hypertension | 2.06 (0.33- 12.68) |
| Active tumor | 2.40 (0.77- 7.47) |
| Chronic pulmonary disease | 0.68 (0.26- 1.82) |
| Stroke | 0.50 (0.14- 1.86) |
| CKD | 0.37 (0.09- 1.65) |
| Presenting vital signs | |
| Fever | 0.22 (0.07- 0.71) |
| Heart rate | 0.97 (0.90- 1.05) |
| Respiratory rate | 1.20 (0.86- 1.67) |
| Systolic blood pressure | 1.11 (0.78- 1.60) |
| Laboratory results |  |
| White blood cell | 1.50 (1.11- 2.03) |
| Hemoglobin concentration | 1.14 (0.89- 1.47) |
| Platelet count | 0.68 (0.52-0.97) |
| hs-CRP | 1.22 (0.88- 1.71) |
| PCT | 0.93 (0.66-1.31) |
| Total bilirubin | 0.32 (0.09-1.17) |
| Blood sugar | 0.85 (0.65- 1.12) |
| Urea nitrogen | 1.42 (0.95- 2.13) |
| Concomitant treatment |  |
| Lopinavir/ritonavir | 6.43 (2.17- 19.07) |

*Note: CKD, chronic kidney disease; hs-CRP, high sensitivity C-reactive protein; PCT, procalcitonin
